# The use of applied improvisation at university: a mini-review

**DOI:** 10.3389/fpsyg.2025.1661912

**Published:** 2026-01-07

**Authors:** Marine Jouin, Isabella Sharvadze, Judit Fekete, Héloïse Longuépée, Hadrien Thomas, Clarisse Grados, Julie De Wever, Maxime Gignon, Mathieu Hainselin

**Affiliations:** 1 CRP-CPO UR 7273, Université Picardie Jules Verne, Amiens, France; 2 Independent Researcher, Konstanz, Germany; 3 Department of Languages for Biomedical Purposes and Communication, Medical School, University of Pécs, Pécs, Hungary; 4 Simulation Center, SimUSanté Epione, CHU Amiens-Picardie, Amiens, France; 5 Department of Preventions, Risks, Medical Information and Epidemiology, CHU Amiens-Picardie, Amiens, France

**Keywords:** applied improvisation, teaching, aptitude, universities, theatrical improvisation, skill

## Abstract

**Aim:**

This mini review aimed to synthesize current evidence on applied improvisation programs in higher education across disciplines, identifying their impact, implementation, evaluation methods, and future research directions.

**Methods:**

Following PRISMA-ScR guidelines, a systematic search was conducted across six databases, covering publications from 1999 to 2024. Inclusion criteria encompassed empirical studies involving university populations engaging in theatrical or applied improvisation interventions. After screening, 54 relevant studies were included. Data extracted included study characteristics, participant demographics, intervention methods, evaluation methodologies, and outcomes.

**Results:**

Most studies originated from the United States (70.4%), targeting students (85.2%), particularly in healthcare education (61.1%). Interventions varied from single workshops to multi-session courses, aiming to improve skills such as communication (62.9%), empathy (25.9%), collaboration (25.9%), confidence (22.2%), and stress management (14.8%). Evaluations often relied on satisfaction surveys or in-house questionnaires, with only 27.8% using standardized tools. Methodological variability and reliance on self-reported measures limited comparability and generalizability of the results.

**Conclusion:**

Applied improvisation demonstrates potential as an innovative educational tool in higher education, enhancing key skills across various disciplines. However, significant methodological limitations—including heterogeneous interventions and assessments—hinder generalization of findings. To advance the field, future research should develop standardized assessment tools, establish consistent intervention protocols, and conduct randomized controlled longitudinal studies to assess the effectiveness and the durability of outcomes. Strengthening interdisciplinary collaborations and employing rigorous research methodologies are essential to optimize the use of applied improvisation in higher education.

## 1 Introduction

Theatrical improvisation (or improv), while often associated with comedy, does not necessarily aim to make people laugh, but to tell a story collaboratively (Fu, 2019). It can be considered an exciting learning environment for training with a defined framework and rules (Hainselin et al., 2017). The use of the tools and exercises of theatrical improvisation for training purposes, beyond an artistic aim, has led to the emergence of applied improvisation.

Applied improvisation is defined as “the use of improvisational theater principles, tools, practices, skills, and competencies in non-theatrical contexts that can lead to personal development, team building, creativity, innovation and/or meaning” [Applied Improvisation Network, cited by Chan et al. (2023)]. This approach follows a structured pedagogical framework: sessions follow a considered progression, alternating practical exercises and debriefing phases, with clearly defined learning objectives (Hoffmann-Longtin et al., 2018).

As noted in Chan et al. (2023) synthesis, the notion of improvisation remains ambiguous. The distinction between theatrical improvisation for artistic purposes and improvisation for professional training purposes is not systematically made explicit in the protocols. This conceptual confusion makes it difficult to assess the specific effectiveness of interventions (Watson, 2011). Some programs favor a purely theatrical approach (Hoffman et al., 2008) while others develop highly specialized protocols such as medical improvisation (De Wever et al., 2023), making it difficult to identify specific mechanisms of action. It is also important to note that the term ‘improvisation’ is also commonly used in the fields of music and the arts. To avoid confusion and loss of data, research into applied improvisation should therefore include the terms applied improvisation, improv and theatrical improvisation.

The pedagogical value of applied improvisation is based on several theoretical foundations. It is part of Kolb’s experiential learning model [Kolb, 1984 cited by Grossman et al. (2021)], in which the learner follows a complete cycle: concrete experience through exercises, reflective observation and conceptualization of learning during debriefing and active experimentation during new exercises. This approach is reinforced by the embodied cognition model, which emphasizes the importance of interactions between cognitive processes, the body, and the environment in learning (Hainselin et al., 2024). In addition, by viewing mistakes as learning opportunities rather than failures, applied improvisation creates a safe environment conducive to risk taking and exploration (Seppänen and Toivanen, 2023). While it shares some characteristics with other experiential learning methods such as simulation or role-play, applied improvisation presents distinctive features (De Wever et al., 2023). It emphasizes spontaneity and adaptation to emerging situations rather than following predetermined scenarios. Rules derived from artistic practice, such as accepting others’ ideas through the “Yes, and …” exercise, listening to others, dealing with uncertainty and being present in the moment, foster a secure and playful learning environment where learners are engaged both cognitively and physically (Bender et al., 2022; Schwenke et al., 2021).

Over the last decade, applied improvisation teaching has developed in higher education, particularly in the health sector, where it is recognized under the term medical improv or Health Professional Training Improv (HPTI) (De Wever et al., 2023). In particular, studies in HPTI show improvements in communication skills and the development of empathy (Gao et al., 2019; Hoffmann-Longtin et al., 2018). However, research is still mostly focused on medical students (Chan et al., 2023), with few studies in other disciplines. Furthermore, there is a lack of a comprehensive synthesis of different initiatives and their impacts across disciplines, which limits our understanding of the effectiveness of this pedagogical tool across higher education.

Although mainly focused on the health sector, existing literature reviews highlight the need for common recommendations to guide research and practice (Chan et al., 2023; Fu, 2019; Gao et al., 2019). The lack of consensus on evaluation methods, the optimal duration of the program, or the choice of exercises hinders the development of a transferable knowledge base between disciplines (Fu, 2019; Gao et al., 2019; Terregino et al., 2019). However, there is no existing review on applied improv in higher education, beyond health.

This review therefore aims to take stock of applied improvisation in higher education across all disciplines and its impact on skills. It also seeks to answer three main questions: Who are the target audiences of applied improvisation programs? How are these programs structured and implemented in higher education? What methods are used to evaluate their impact? This analysis will enable identifying good practices and propose recommendations for standardizing future research in this area.

## 2 Methodology

### 2.1 Searching strategy

In our methods, we follow the PRISMA-ScR recommendations (Tricco et al., 2018). The protocol, established in June 2024 and revised in September 2024, was registered before the start of the Open Science Framework (OSF) study.

The literature search covered the period 1999–2024. We chose 1999 as the starting date because this year marked the Bologna process in European higher education which emphasized the quality of learning and transversal competencies (European Commission, 2022). Six databases are selected because they are the most commonly used and for their institutional access: PubMed and via EBSCO HOST: Academic Search Elite, APA PsycArticles, APA PsycInfo, Psychology and Behavioral Sciences Collection, and Business Source Complete.

The search equation, developed jointly by two authors (MJ and MH) and validated by a pilot study (available on OSF), was: (improvisation OR improv OR ‘improvisational theater’ OR improvisational OR ‘theatrical improvisation’ OR ‘medical improvisation’ OR ‘applied improvisation’ OR ‘medical improv’ OR HPTI) AND (teaching OR training OR learning OR using OR skill* OR competence) AND (student* OR education OR formation OR university) NOT (music OR jazz OR dance).

### 2.2 Selection criteria

The criteria were structured according to the PCC format: population (university population: students/teachers), concept (theatrical/applied improvisation), context (higher education training).

Inclusion criteria encompassed empirical studies involving university populations engaging in theatrical or applied improvisation interventions.

Exclusion criteria included: (1) musical/dance improvisation, (2) inaccessible full text, (3) theoretical articles/recommendations/literature reviews.

### 2.3 Selection and analysis process

Selection and analysis were carried out in two distinct phases. In the selection phase, two authors (MJ and IS) independently screened the titles and abstracts of the identified articles. In disagreement, a third researcher (MH) was consulted to reach a consensus. Full texts were consulted if relevance was not apparent from the abstract.

The same authors (MJ and IS) independently read the selected articles for the analysis phase. Data were extracted using a standardized form and arranged by theme in an Excel spreadsheet, including study characteristics (authors, year, country, design), participant characteristics (type, discipline, level of study), intervention methods (type of improvisation, duration, frequency), evaluation methodology (tools, timeframe) and main outcomes. If full texts were unavailable, the corresponding authors were contacted and given 1 month to respond. Articles that remained inaccessible after this procedure were excluded from the analysis.

### 2.4 Results of article selection process

**Figure 1:**
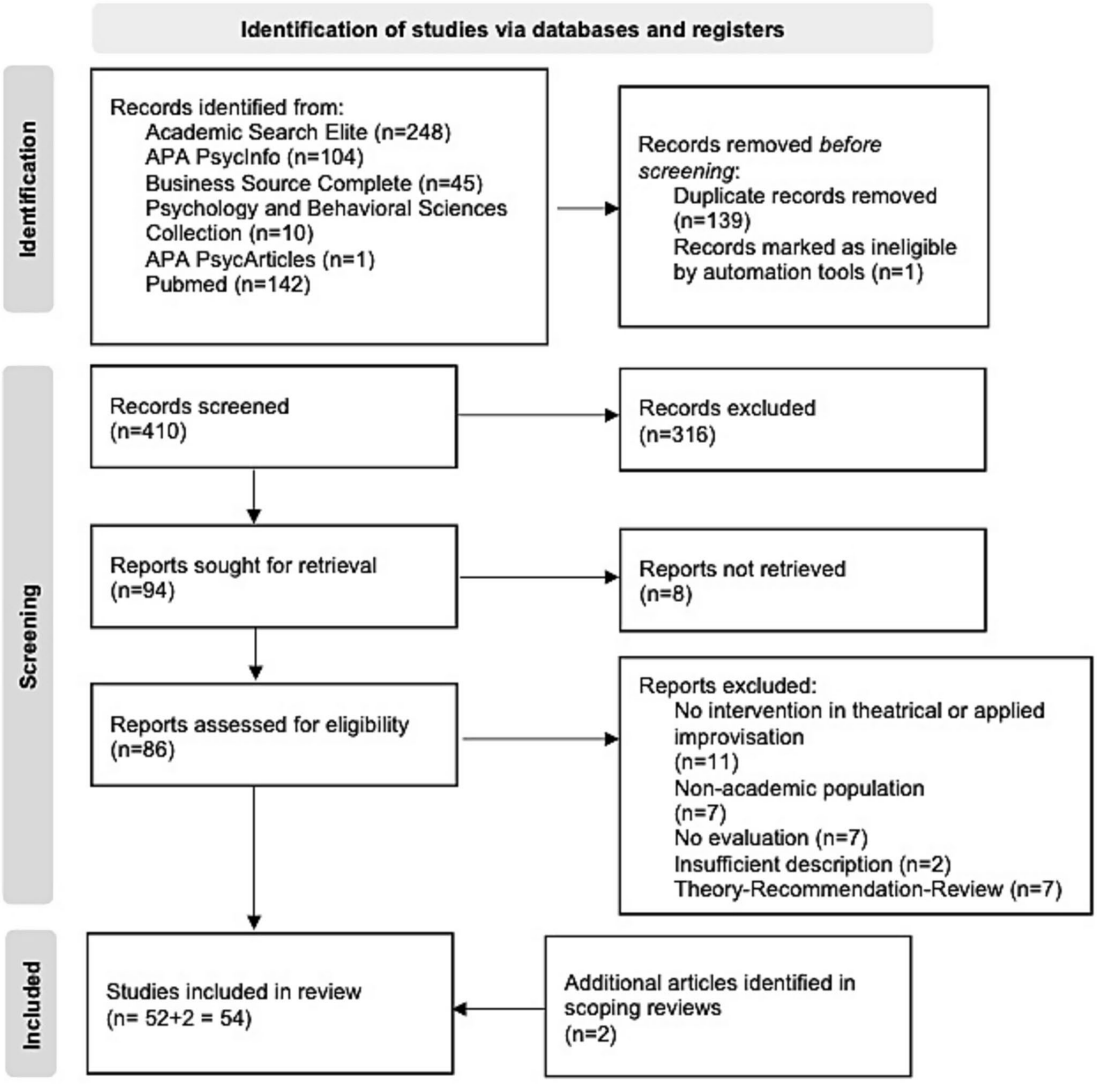
PRISMA flow diagram for article selection.

The initial database search identified 550 articles, of which 410 were retained for abstract review after deduplication. Of these, 316 were excluded because they did not meet the inclusion criteria. Of the remaining 94 articles, 8 could not be retrieved despite attempts to contact the authors. Analysis of the 86 full texts led to the exclusion of 34 articles: 11 did not involve theatre or applied improvisation, 7 involved a non-academic population, 7 did not provide an evaluation, 2 provided an inadequate description of the intervention, and 7 were theoretical articles or literature reviews. We compared the included articles with articles from previous literature reviews (Chan et al., 2023; Fu, 2019; Gao et al., 2019). Identifying 2 additional articles through literature reviews brought the final number of articles included to 54 (Figure 1).

## 3 Discussion

This mini-review combines results and discussion sections to provide an analysis of our findings in direct relation to our research questions. For detailed data on individual studies, readers are referred to Supplementary materials.

### 3.1 Study characteristics and target populations

#### 3.1.1 Geographic origin and population

Most studies were from the United States (70%, *n* = 38), followed by European countries (17%, *n* = 9). Other regions were poorly represented (9%, *n* = 5).

Most programs targeted students (85%, *n* = 46), predominantly in healthcare (61%, *n* = 33), with 27 studies focusing on medical students. This concentration in medicine likely reflects the high uncertainty inherent to clinical practice (Fu, 2019). Other disciplines represented include management (9%, *n* = 5), education (7%, *n* = 4), and various fields (engineering, library science, clinical social work, sport; 9%, *n* = 5). The predominance of healthcare suggests applied improvisation may be particularly effective in high-uncertainty professional contexts requiring rapid adaptation and decision-making under pressure. Furthermore, the majority of health studies (*n* = 26) focused on the impact of improvisation on communication, which is an essential skill in clinical practice (Chan et al., 2023).

The median sample size was 38 participants (min 5, max 494), and only 5 studies included a control group.

A table detailing the distribution of target audiences by discipline and the associated intervention methods is available in Supplementary Table 2.

#### 3.1.2 Impact on different disciplines

Health studies show results in terms of improved interpersonal skills, with increases in empathy scores (Amjadi et al., 2024; Ballon et al., 2007; Schwartz et al., 2024) and communication skills (Boesen et al., 2009; Hoffman et al., 2008; Shochet et al., 2013; Tetenbaum-Novatt and Alexander, 2023). Improvisation in higher education also promotes the development of adaptive skills, such as tolerance for uncertainty (Felsman et al., 2020) and divergent thinking (Mourey, 2020), while helping to improve occupational well-being (Neel et al., 2021; Westcott et al., 2023) and reduce social anxiety (Hu et al., 2024). Beyond the medical education field, positive effects can be seen in other disciplines. Applied improvisation enables future managers to develop greater flexibility in their approach to leadership (Paquelet Moreira et al., 2022). In education, applied improvisation improves the management of classroom interactions with mathematics teachers who have developed thought patterns for dealing with unexpected moments (Morales-Almazan, 2022). The approach has also proved beneficial in language learning, with a significant increase in self-esteem and a reduction in language anxiety among student teachers of English (Zondag, 2021).

### 3.2 Implementation and structure of workshops/programs

#### 3.2.1 Formats and length of intervention

Long interventions (>1 session) accounted for 59% of studies (*n* = 32), with a variable duration of 4 to 12 sessions. Single workshops (41%, *n* = 22) lasted 1 to 3 h. Participation was voluntary in 46% of cases (*n* = 25) and compulsory in 30% (*n* = 16).

Programs vary in length and intensity, ranging from single 45-min sessions (Grossman et al., 2021) to semester-long programs of more than 15 h (Zelenski et al., 2020). There is also considerable variability in sample size, ranging from 5 participants (Bing-You et al., 2018) to 494 students (Fessell et al., 2020). This variability reflects the diversity of educational objectives and institutional constraints.

Applied improvisation’s benefits appear to be greater when it is coherently integrated into the curriculum (Huffaker and West, 2005; Lawrence and Coaston, 2017). Integration over a semester enabled management students to understand better leadership dynamics (Huffaker and West, 2005). By developing a medical improvisation program around three pedagogical axes: presence, acceptance, and trust, Bender et al. (2022) demonstrate a significant improvement in interprofessional communication. These themes are consistent with Chan et al. (2023) recommendations for the essential skills in medical improvisation.

#### 3.2.2 Teaching structure

The pedagogical structure of improvisation in higher education varies in terms of the profiles of the trainers and the methods used. The most common model is the pairing of a subject teacher and an improvisation expert (Watson, 2011; Westcott et al., 2023). Several studies have highlighted the value of interdisciplinary collaboration (De Wever et al., 2023; Donovan et al., 2020; Kaplan-Liss et al., 2018; Paquelet Moreira et al., 2022) in promoting a global pedagogical approach and bringing together different areas of expertise, particularly during the debriefing phase. However, we still need specific studies on the impact of such teaching model to have a definitive answer on what is the best configuration.

The structured framework of applied improvisation around core values such as collaboration, adaptability and trust create a safe and supportive learning environment, allowing open dialogue on sensitive social issues, such as racism in healthcare (Ayub et al., 2024). However, the trainer must play a central role in maintaining this environment, balancing the stress inherent in improvisational exercises with the development of targeted skills (Temezhnikova and Bazarov, 2020). This requires fostering trust while carefully calibrating the instructions provided. Overly prescriptive guidance can inhibit the spontaneity and openness essential for effective learning (Archieri, 2022). Although rooted in fiction, improvisation often evokes genuine emotional responses, underscoring its potential to deeply engage participants (Seppänen et al., 2020).

The teaching progression should be structured by choosing exercises and moving from basic exercises to more complex professional situations (Tetenbaum-Novatt and Alexander, 2023). The ‘Yes, and …’ principle forms the basis of many programs (Kaplan-Liss et al., 2018; Watson, 2011). This principle encourages active listening and collaborative construction, essential professional interaction skills (Shochet et al., 2013). Sessions typically begin with joint warm-up exercises: mirror exercises to develop mutual observation, an emotion circle to work on expressing and recognizing affect (Hu et al., 2024), and group exercises such as ‘Group Think’ or ‘That’s That’ to develop cohesion (Lawrence and Coaston, 2017). Hoffmann-Longtin et al. (2018) highlight the importance of explicitly aligning the chosen exercises with the learning objectives and intended professional context.

#### 3.2.3 Implementation challenges

The association of applied improvisation with other teaching methods has been found in numerous studies (De Wever et al., 2023; Grossman et al., 2021; Hobson et al., 2019; Hu et al., 2024; Kukora et al., 2020). In healthcare, medical improvisation can be combined with clinical simulation (De Wever et al., 2023) or objective structured clinical examinations (OSCE) (Grossman et al., 2021; Terregino et al., 2019). The advantage of combining this with simulation would be assessing the impact on actual clinical performance and, thus, the skills’ durability (Shochet et al., 2013). In addition, improvised role-playing would require fewer logistical resources than standardized patients while improving communication (Boesen et al., 2009; Kukora et al., 2020).

However, there are a few challenges to consider when implementing improvisation. Recruitment and retention of participants is difficult, particularly in longitudinal studies (Li et al., 2022). Logistical and institutional constraints have a significant impact on the implementation of programs. The need for suitable facilities, time constraints on already busy training schedules, and limited financial resources are recurring barriers (Westcott et al., 2023). The pandemic has exacerbated these difficulties, forcing rapid adaptation to virtual formats whose comparative effectiveness has yet to be established (Amjadi et al., 2024).

### 3.3 Evaluation and impact

#### 3.3.1 Assessment approaches and tools

Most studies used satisfaction questionnaires (37%, *n* = 20) or in-house questionnaires with Likert scales or open questions (15%, *n* = 8). Fifteen studies (27.8%) used standardized tools: Jefferson Scale of Empathy (JSPE), CARE Measure, Interpersonal Reactivity Index (IRI), Interprofessional Collaborative Competence Attainment Survey (ICCAS), Clinical Communication Skills Questionnaire (SE-12), Uncertainty Tolerance Scale (UTS), Empathic Communication Coding System (ECCS), Social Anxiety Scale (SAS), Intentional Self-Regulation (ISR), Aesthetic Experience Scale (AES), Reading the Mind in the Eyes Task (RMET). The others used interviews (13%, *n* = 7) or observations (6%, *n* = 3).

The most commonly targeted skills were communication (63%, *n* = 34), confidence (22%, *n* = 12), empathy (26%, *n* = 14), collaboration (26%, *n* = 14) and stress management (15%, *n* = 8).

The assessment tools used vary according to the objective and discipline. In healthcare, several standardized scales are used: the Interpersonal Reactivity Index (IRI) and the Jefferson Scale of Empathy for measuring empathy (Amjadi et al., 2024; Schwartz et al., 2024), the Empathic Communication Coding System for communication (Grossman et al., 2021) and the Consultative And Relational Empathy (CARE) measure for relational empathy (Zelenski et al., 2020). Qualitative approaches include reflective diaries, semi-structured interviews, and taped debriefings (Li et al., 2022; Paquelet Moreira et al., 2022; Perrmann-Graham et al., 2022). Some studies use a mixed approach, such as Seppänen et al. (2020), which combines psychophysiological measures (ECG, EEG, EDA, facial EMG) with self-reports of stress. Nevertheless, the majority of assessments are still primarily based on self-report (Ayub et al., 2024; De Wever et al., 2023; Donovan et al., 2020; Erdman and Dellasega, 2024), underlining the need to develop more hetero-report assessment tools (such as observation grids like ACT4Ethics, Daboval et al., 2019), especially to measure the transfer of skills in real-life situations and their maintenance over time (Shochet et al., 2013).

#### 3.3.2 Durability of effects

The majority of studies (28%, *n* = 15) used only post-intervention measures. Only 19% (*n* = 10) used a pre-post design and 15% (*n* = 8) included medium to long-term follow-up (pre-post with remote follow-up between 1 and 8 months).

The durability of the effects remains uncertain. Some studies find regression to baseline after 3 to 8 months for short interventions (Cai et al., 2019; Minow et al., 2024; Romanelli and Tishby, 2019). However, longer programs show better long-term skills retention (Mourey, 2020). The positive effects of a 10-week program with marketing students on collaboration and feelings of self-efficacy were maintained at 4 months (Mourey, 2020). These differences in the durability of effects raise questions about the relevance of short versus long training courses (Del Vecchio et al., 2022; Schwartz et al., 2024; Zelenski et al., 2020) and highlight the need for longitudinal studies (Rice-Bailey, 2021).

The findings underscore the potential of applied improvisation to address critical gaps in medical and healthcare education, particularly in fostering communication and teamwork skills (Bender et al., 2022). Despite positive learner feedback, the heterogeneity in curriculum design and assessment methods highlights the need for standardization. Drawing from experiential learning theories, future efforts should focus on aligning applied improvisation exercises with clear learning objectives and developing robust evaluation frameworks (Hoffmann-Longtin et al., 2018). Additionally, expanding facilitator training to include interdisciplinary expertise could enhance program scalability and impact (Zelenski et al., 2020).

### 3.4 Limitations

Our review has several limitations that deserve mention.

Research was limited to two languages (French and English), potentially excluding relevant studies published in other languages. Only databases available through PubMed and EBSCOhost interfaces were consulted.

Certain limitations are inherent in the review methodology itself. Unlike a systematic review, this approach does not allow for a formal assessment of the methodological quality of the included studies, which may affect the robustness of the conclusions. The breadth of the field of investigation may result in a lack of depth in the analysis of certain specific aspects, such as variations in intervention formats or application contexts. The inclusion of studies with different methodologies (qualitative, quantitative, mixed) enriches the overall understanding, but complicates the synthesis of results and may mask important methodological differences. This heterogeneity of evaluation methods makes it particularly difficult to directly compare results across studies.

### 3.5 Perspectives

The findings of this review identify several key areas for further development in research into the application of improvisation in higher education. In addition to the limitations identified, there are opportunities to enhance the methodological rigor of future studies, standardize assessment practices and deepen our understanding of learning mechanisms, including the cognitive processes involved (Krueger et al., 2025). A systematic review focusing on the efficacy of applied improvisation interventions, particularly within the healthcare sector where there is a wealth of existing literature, would facilitate the establishment of more robust evidence levels.

Table 1 summarizes the main recommendations and suggests concrete avenues for their implementation.

**Table 1:** Recommendations for setting up an improvisation protocol.

Themes	Limits	Recommendations
Methodology	Heterogeneity in intervention protocols (duration, sample size) making the comparison and the generalization of results difficult	Establish standardized intervention protocols: accurate documentation of exercises and their progression would facilitate the reproducibility of studies (Chan et al., 2023)
Measures	Predominance of self-reported measures, compromising external validity	Use validated psychometric instruments (Fu, 2019; Gao et al., 2019) Develop evaluation tools specific to improvisation (Terregino et al., 2019)
Experimental design	Lack of control groups and randomization limiting the validity of conclusions	Use of randomized controlled trials with control groups
Conceptualization	Lack of explicit objective, insufficient description of the program and exercises Variability in the pedagogical progression of workshops	Clearly define pedagogical objectives, explicitly link exercises to targeted professional skills Establish consensus standards for exercise selection and development (Chan et al., 2023) Step-by-step approach with basic exercises (Minow et al., 2024) including systematic debriefing time (Westcott et al., 2023)
Qualification of improv facilitator	Limited trainer qualification	Form multidisciplinary teaching teams combining improvisation expertise and disciplinary competence (De Wever et al., 2023)
Follow up	Lack of long-term effect evaluation and understanding of skill maintenance mechanisms	Conduct longitudinal studies (at least 3-month follow-up), study skills retention mechanisms, evaluate the impact of refresher sessions (Romanelli and Tishby, 2019)
Cultural differences	Some exercises may not be suitable for all cultural contexts, particularly regarding status dynamics and attitudes toward failure	Clarify and integrate cultural differences in program design, adapt exercises to local norms and attitudes, consider cultural specificities in evaluation (Henrich et al., 2010)
Collaboration	Research isolation by discipline limiting comprehensive understanding of learning processes through improvisation	Strengthen interdisciplinary collaborations, develop networks of researchers and practitioners, facilitate sharing of best practices (De Wever et al., 2023)

## 4 Conclusion

This first review demonstrates the potential of improvisation as an innovative pedagogical tool in higher education, highlighting the methodological and practical challenges that must be overcome. The studies reviewed show significant positive effects in various areas, including health, management, and teacher training. Improvisation improves empathy, communication, error management, and teaching flexibility. However, the levels of evidence are not the same.

The pedagogical framework is progressive, from developing basic skills to specific professional applications. However, methodological limitations such as the variability of the protocols, the ambiguity between the theoretical concepts of theatrical and applied improvisation, and the predominance of self-assessment limit the generalizability of the conclusions.

Randomized controlled trials using validated psychometric instruments are needed to better understand the links between improvisation principles and skills development. The development of standardized protocols and longitudinal impact assessment will facilitate the broader adoption of this approach in higher education.
