# Supplementary material for: The use of applied improvisation at university: a mini-review
Source: Front Psychol. 2026 Jan 7;16:1661912. doi: 10.3389/fpsyg.2025.1661912 (PMC12819688; doi:10.3389/fpsyg.2025.1661912)
Supplement: Supplementary file 1 [file Data_Sheet_1.PDF]

Supplementary Table : Characteristics of included studies

Title	First author	Year	Country	Discipline	Target population	Control group	Intervention	Skills	Evaluation	Time of evaluation	Exercise description	Trainer profile	References
Zoom Improv is accessible and enhances medical student empathy	Amjadi	2024	USA	medicine	Students	Yes	One-off workshop / 90 minutes	Empathy	Jefferson Scale of Empathy (JSPE)<br>CARE Measure<br>Interpersonal Reactivity Index	Pre / Post	No	Not mentionned	Amjadi, M. F., Kociubuk, J., Hollnagel, F., Tsenkova, V. K., & Zelenski, A. B. (2024). Zoom Improv is accessible and enhances medical student empathy. BMC Medical Education, 24(1), 1049. https://doi.org/10.1186/s12909-024-06017-6
Évaluer des compétences : De l'intention des enseignants au vécu des étudiants. Le cas d'un atelier d'improvisation.	Archieri	2022	France	STAPS	Students	No	Programme/8 sessions, total of 24 hours	Interaction with others, Letting go, Speaking out	Post interviews<br>Satisfaction questionnaire	Last session	Not completely	Not mentionned	ARCHIERI, C. (2022). Évaluer des compétences : De l'intention des enseignants au vécu des étudiants. Le cas d'un atelier d'improvisation. Revue Education et Socialisation, 64, 1-13. afh.
Theater for Healthcare Equity; A Model for Inclusion and Anti-Bias Training in Academic Medicine	Ayub	2024	USA	medicine	Students, Medical staff	No	Programme/8 sessions of 90 minutes over 2 days (17-18 November 2022)	Anti-racist skills	In house questionnaire	Post	No	External expert facilitators	Ayub N, Regalia C, Wilson T, Gaughf C, Anderson C, Banerjee D. Theater for Healthcare Equity; A Model for Inclusion and Anti-Bias Training in Academic Medicine. R I Med J (2013). 2024 Jun 3;107(6):29-34. PMID: 38810013.
Headspace Theater:<br>An Innovative Method for Experiential Learning of Psychiatric Symptomatology Using Modified Role-Playing and Improvisational Theater Techniques	Ballon	2007	Canada	medicine	Students, Medical staff	No	Programme/14 sessions of 50 minutes	Empathy	Satisfaction questionnaire	Post	Role-playing: scenario described	Expert facilitators trained	Ballon BC, Silver I, Fidler D. Headspace theater: an innovative method for experiential learning of psychiatric symptomatology using modified role-playing and improvisational theater techniques. Acad Psychiatry. 2007 Sep-Oct;31(5):380-7. doi: 10.1176/appi.ap.31.5.380. PMID: 17875623.
Improving interprofessional communication: Conceptualizing, operationalizing and testing a healthcare improvisation communication workshop	Bender	2022	USA	medicine, public health, nursing	Students	No	One-off workshop/3h	Communication, inter-professional collaboration	Interprofessional Collaborative Competence Attainment Survey (ICCAS)<br>Clinical Communication Skills Questionnaire (SE-12)	Pre / Post	No	Improvisation expert and healthcare expert	Bender, M., Veenstra, J., & Yoon, S. (2022). Improving interprofessional communication : Conceptualizing, operationalizing and testing a healthcare improvisation communication workshop. Nurse Education Today, 119, 105530. https://doi.org/10.1016/j.nedt.2022.105530
Using improvisation to promote teaching- and thinking-in-action	Bing-You	2018	USA	medicine	Teachers	No	Programme/4sessions, total of 9h	Mindfulness	Fribourg Mindfulness Inventory<br>Mindfulness in Teaching Scale<br>Qualitative follow-up interviews	Pre / Post	Yes<br>- "One-Word Story"<br>- "Machines"<br>- "ABC Story"<br>- "Yes, and..."<br>- "Authorities"<br>- "Instant Replay"	Not mentionned	Bing-You, R., White, P., Dreher, G., & Hayes, V. (2018). Using improvisation to promote teaching- and thinking-in-action. Medical Education, 52(5), 566-567. psyh. https://doi.org/10.1111/medu.13553
Improvisational Exercises to Improve Pharmacy Students' Professional Communication Skills	Boesen	2009	USA	pharmacy	Students	No	Programme/6 sessions of 2h, total of 12h	Communication	Professional scenarios :<br>Standardised patient examinations (SPE)	Pre / Post / Longitudinal follow-up (3 years)	Yes<br>"Yes and"	Trained instructors with experience in improvisation	Boesen KP, Herrier RN, Apgar DA, Jackowski RM. Improvisational exercises to improve pharmacy students' professional communication skills. Am J Pharm Educ. 2009 Apr 7;73(2):35. doi: 10.5688/aj730235. PMID: 19513173; PMCID: PMC2690892.
Can I Get a Suggestion? Medical Improv as a Tool for Empathy Training in Obstetrics and Gynecology Residents	Cai	2019	USA	medicine	Students	No	One-off workshop/ 60 minutes	Communication, Empathy	Jefferson Scale of Empathy (JSPE)	2 weeks before the procedure<br>Immediately after<br>At 1 month<br>At 3 months<br>At 6 months	Yes<br>"So This Morning, Right?", "Yes, And...", "Scene Painting", "Late to Work"	A trained simulation specialist and professional improvisation actress	Cai F, Ruhotina M, Bowler M, Howard E, Has P, Frishman GN, Wohlrab K. Can I Get a Suggestion? Medical Improv as a Tool for Empathy Training in Obstetrics and Gynecology Residents. J Grad Med Educ. 2019 Oct;11(5):597-600. doi: 10.4300/JGME-D-19-00185.1. PMID: 31636832; PMCID: PMC6795330.
Medical Education Empowered by Theater (MEET)	De Carvalho	2020	Bresil	medicine	Students	No	Not mentioned	Communication, Empathy, Interpersonal relations	Jefferson Scale of Empathy (JSPE)<br>Satisfaction questionnaire	Pre / Post	Yes<br>In the supplemental digital appendix	2 actors + 2 teaching doctors	de Carvalho Filho, M. A., Ledubino, A., Frutuoso, L., da Silva Wanderlei, J., Jaarsma, D., Helmich, E., & Strazzacappa, M. (2020). Medical Education Empowered by Theater (MEET). Academic Medicine: Journal of the Association of American Medical Colleges, 95(8), 1191-1200. https://doi.org/10.1097/ACM.0000000000003271
Acting to teach communication skills to nurses	del Vecchio	2022	USA	nursing care	Students	No	Programme/2 workshops from 30 to 90 minutes	Communication, active listening, collaboration, flexibility, emotions	Questionnaires with 5-point Likert scale<br>Written feedback from participants	Post each session	Yes<br>In the table of the article	Creator of the course (former actor) and a teacher without theater training	Del Vecchio, A., Moschella, P. C., Lanham, J. G., & Zavertnik, J. E. (2022). Acting to teach communication skills to nurses. The Clinical Teacher, 19(4), 289–293
Medical improvisation helps speech therapists to improve their communication skills	De Wever	2023	France	speech therapy	Students	No	Not mentioned	Communication	Satisfaction questionnaire	Post	Not completely<br>"Yes and ?"	Theatrical improvisation trainer	De Wever J, Gignon M, Hainselin M. Medical improvisation helps speech therapists to improve their communication skills. Med Educ. 2023;57(2):189-190. doi:10.1111/medu.14983
Applied improvisation and transdisciplinary simulation: a necessity for any health curriculum?	De Wever	2023	France	medicine, speech therapy, midwifery, nursing	Students	No	Programme/16h of training	Communication	Satisfaction questionnaire	Post	No	A professional improvisation facilitator<br>A transdisciplinary team including:<br>- Health professionals<br>- Psychologists<br>- Theater teachers<br>- Simulation center staff	De Wever J, Hainselin M and Gignon M (2023) Applied improvisation and transdisciplinary simulation: a necessity for any health curriculum? Front. Med. 10:1237126. doi: 10.3389/fmed.2023.1237126
An Applied Improvisational Pharmacy Communication Workshop Implemented During Orientation for First-Year Pharmacy Students	Donovan	2020	USA	pharmacy	Students	No	One-off workshop/2h boot camp	Communication	Satisfaction questionnaire	Post	Yes (in the annex)<br>"Icebreaker"<br>"Enemy/Protector"<br>"Time traveler"<br>"Zip-zap-zop"<br>"Beaker/Bagel"	Multidisciplinary team:<br>- Full professor in communications<br>- Post-doctoral fellow in communication<br>- 4 doctoral students in communication<br>- Clinical professor of pharmacy	Donovan, E. E., Brown, L. E., Rush, S. K., Greenwell, M. R., Table, B., Zhu, Y., & Kearns, K. D. (2020). An Applied Improvisational Pharmacy Communication Workshop Implemented During Orientation for First-Year Pharmacy Students. American Journal of Pharmaceutical Education, 84(1), 7250. https://doi.org/10.5688/ajpe7250
Using improvisation to enhance communication skills in 4th year medical students	Erdman	2024	USA	Medicine	Students	No	Programme/eight 2.5-hour sessions	Communication, Empathy, Active listening, Collaboration, Well-being	Satisfaction questionnaire<br>Oral and written feedback from participants	During / Post	Not completely	Not mentionned	Erdman, L., & Dellasega, C. (2024). Using improvisation to enhance communication skills in 4th year medical students. Medical Education. https://doi.org/10.1111/medu.15503
Improv experience promotes divergent thinking, uncertainty tolerance, and affective well-being	Felsman	2020	USA	/	Students	Yes	One-off workshop/20 minutes	well-being, divergent thinking	Alternative Uses Task (AUT) for divergent thinking<br>Uncertainty Tolerance Scale (UTS)	Pre / Post	Yes	Advanced students without improvisation experience	Felsman, P., Gunawardena, S., & Seifert, C. M. (2020). Improv experience promotes divergent thinking, uncertainty tolerance, and affective well-being. Thinking Skills and Creativity, 35. psyh. https://doi.org/10.1016/j.tsc.2020.100632
Medical Improvisation Training for all Medical Students: 3-Year Experience	Fessel	2020	USA	medicine	Students	No	One-off workshop/ between 2h30 and 3h	Communication	Satisfaction questionnaire	Post / at 3 months (2016)	Yes<br>"The Rant"<br>"Mirroring"<br>"Time Traveler"<br>"Ta-Da"<br>"Drawing game"	Alan Alda Center trainers (2015) then local instructors (2016-2017)	Fessell, D., McKean, E., Wagenschutz, H., Cole, M., Santen, S. A., Cermak, R., Zurales, K., Kukora, S., Lantz-Gefroh, V., Kaplan-Liss, E., & Alda, A. (2020). Medical Improvisation Training for all Medical Students : 3-Year Experience. Medical Science Educator, 30(1), 87-90. https://doi.org/10.1007/s40670-019-00885-0
Improv to improve medical student communication	Grossman	2021	USA	medicine	Students	Yes	One-off workshop/45 minutes followed immediately by an activity with a standardised patient	Communication, Empathy	Empathic Communication Coding System (ECCS)<br>Patient satisfaction questions derived from the Press Ganey™ and Hospital Consumer Assessment of Healthcare Providers and System Surveys™.	At the end of each part of the interaction	Yes in additional material<br>"Wish-Whoosh"<br>"Gifts"<br>"So what you're saying"	Theater teachers and doctors	Grossman, C. E., Lemay, M., Kang, L., Byland, E., Anderson, A. D., Nestler, J. E., & Santen, S. A. (2021). Improv to improve medical student communication. The Clinical Teacher, 00, 1-6. https://doi.org/10.1111/tct.13336
Telling the Patient's Story: using theatre training to improve case presentation skills	Hammer	2011	USA	medicine	Students	No	Programme/10 sessions in 1 week, 25 hours total contact time (10 sessions)	Communication, Speaking	In-house questionnaire (Likert scale / free response)	Pre / Post	Yes in the table of the article	Theater teachers and doctors	Hammer, R. R., Rian, J. D., Gregory, J. K., Bostwick, J. M., Barrett Birk, C., Chalfant, L., Scanlon, P. D., & Hall-Flavin, D. K. (2011). Telling the Patient's Story: Using theatre training to improve case presentation skills. Medical Humanities, 37(1), 18-22. https://doi.org/10.1136/jmh.2010.006429
Improving medical student communication skills through improvisational theatre	Hoffman	2008	USA	medicine	Students	No	Programme/ one semester	Communication, Interpersonal relations, Self-confidence, Active listening	In house questionnaire (5-point scale)	Post	Not completely	Improvisation experts and doctors	Hoffman, A., Utley, B., & Ciccarone, D. (2008). Improving medical student communication skills through improvisational theatre. Medical Education, 42(5), 537-538. https://doi.org/10.1111/j.1365-2923.2008.03077.x
Teaching Advocacy Communication to Pediatric Residents: The Efficacy of Applied Improvisational Theater (AIT) as an Instructional Tool	Hoffman-Longtin	2018	USA	Paediatrics	Students		One-off workshop/7h	Communication, Empathy, Interpersonal relations	In-house questionnaire (Likert scale / free response)	Pre/post/Follow-up: 6-12 months after	Yes<br>- Improvisation for Physicians<br>- Distilling Your Message<br>- Partnering with the Community<br>- Media Training	Not mentionned	Hoffmann-Longtin, K., Organ, J. M., Helphinstine, J. V., Reinoso, D. R., Morgan, Z. S., & Weinstein, E. (2018). Teaching advocacy communication to pediatric residents: the efficacy of applied improvisational theater (AIT) as an instructional tool. Communication Education, 67(4), 438–459. https://doi.org/10.1080/03634523.2018.1503314
Active Learning on Center Stage: Theater as a Tool for Medical Education	Hobson	2019	USA	medicine	Teachers	No	One-off workshop/75 minutes	Communication	In house questionnaire	At 5 months post	Yes (in the annex)	Improvisation experts	Hobson, W. L., Hoffmann-Longtin, K., Loue, S., Love, L. M., Liu, H. Y., Power, C. M., & Pollart, S. M. (2019). Active Learning on Center Stage: Theater as a Tool for Medical Education. MedEdPORTAL, 15, 10801. https://doi.org/10.15766/mep_2374-8265.10801
The change in aesthetic experience and empathic concern predicts theory of mind ability: Evidence from drama improvisation training	Hu	2024	Chine	not communicated (not in arts)	Students	No	Programme/6 weeks, one 60-minute session per week	social anxiety, well-being	Social Anxiety Scale (SAS)<br>Interpersonal Reactivity Index (IRI)<br>Intentional Self-Regulation (ISR)<br>Aesthetic Experience Scale (AES)<br>Reading the Mind in the Eyes Task (RMET)	Pre/post	Yes	2d author, trained in applied improvisation	Hu, Y., Li, R., Jiang, X., & Chen, W. (2024). The change in aesthetic experience and empathic concern predicts theory of mind ability: Evidence from drama improvisation training. The Arts in Psychotherapy, 89, 102167. https://doi.org/10.1016/j.aip.2024.102167
Enhancing Learning in the Business Classroom: An Adventure with Improv Theater Techniques	Huffaker	2005	USA	management	Students	No	Programme/1 academic term (10 weeks)	creativity, leadership	Instructor observations<br>in-house self-assessment	During / mid-term/post/ feedback 7 months after the course	Yes	2 authors	Huffaker, J. S., & West, E. (2005). Enhancing learning in the business classroom: An adventure with improv theater techniques. Journal of Management Education, 29(6), 852-869. https://doi.org/10.1177/1052562905277311
Teaching Medical Students to Communicate With Empathy and Clarity Using Improvisation	Kaplan-Liss	2018	USA	dentistry, medicine, nursing	Students	No	Programme/6 weekly sessions of three hours each (18 hours in total)	Communication, Empathy, Active listening	Course evaluation on a 1-5 point scale	Post	Yes<br>"Mirroring"<br>"Yes and"<br>Role play	Improvisation expert and doctor	Kaplan-Liss, E., Lantz-Gefroh, V., Bass, E., Killebrew, D., Ponzio, N. M., Savi, C., & O'Connell, C. (2018). Teaching medical students to communicate with empathy and clarity using improvisation. Academic Medicine, 93(3), 440-443. https://doi.org/10.1097/ACM.0000000000002031
Hilariously Bad News: Medical Improv as a Novel Approach to Teach Communication Skills for Bad News Disclosure	Kukora	2020	USA	medicine	Doctoral students	No	One-off workshop/3 hours	Communication, Interaction with others, Interpersonal relations, Active listening	In house questionnaire (Likert scale 5 or 7 points ; Yes/no questions / Free response)	Pre/post/at 6 months	Yes	Not mentionned	Kukora, S. K., Batell, B., Umoren, R., Gray, M. M., Ravi, N., Thompson, C., & Zikmund-Fisher, B. J. (2020). Hilariously Bad News: Medical Improv as a Novel Approach to Teach Communication Skills for Bad News Disclosure. Academic Pediatrics, 20(6), 879-881.
Whose Line Is It, Anyway? Using Improvisational Exercises to Spark Counselor Development	Lawrence	2017	USA	consulting	Students	No	One-off workshop/2h45	Communication, collaboration, risk-taking, spontaneity	Qualitative debriefing questions for each exercise	during	Yes<br>Group Think<br>That's That<br>Storytellers<br>Perception Part<br>Inside My Head<br>Heaven Is a Place on Earth	University teacher-researchers specialized in counseling	Lawrence, C., & Coaston, S. C. (2017). Whose Line Is It, Anyway? Using Improvisational Exercises to Spark Counselor Development. Journal of Creativity in Mental Health. https://doi.org/10.1080/15401383.2017.1281185

Supplementary Table : Characteristics of included studies

Exploring a brief medical improvisational performing arts intervention for genetic counseling graduate students	Li	2022	USA	genetic counselling, medicine	Students, New graduates	No	One-off workshop/2 hours	Adaptability, Communication, Self-confidence, Stress management	Intervention questionnaires (4-point Likert scale)<br>Semi-structured interviews	Pre/Post/at 2 months	Yes in the table of the article	1st author, trained in improv	Li, W., Scherr, C. L., Fenter, R. B., Watson, K. L., & Wicklund, C. A. (2022). Exploring a brief medical improvisational performing arts intervention for genetic counseling graduate students. Journal of Genetic Counseling, 31(5), 1193-1205. https://doi.org/10.1002/jgc4.1590
Good ideas for teaching: Design and implementation of the communication workshop "me as team member" for third-year medical students	Minow	2024	Germany	medicine	Students	No	One-off workshop/ (9am-5.30pm)	Communication, Self-confidence, Stress management, Interprofessional collaboration, Error management	ISVS (Interprofessional Socialization and Valuing Scale)<br>EOQ (Error Orientation Questionnaire)<br>Satisfaction questionnaire<br>Institutional assessments	Pre/Post/at 8 months	Not completely	Two external actresses and communications trainers	Minow, A., Gandras, K., Wagner, J., & Westermann, J. (2024). Good ideas for teaching: Design and implementation of the communication workshop "me as team member" for third-year medical students. GMS Journal for Medical Education, 41(3), Doc25. https://doi.org/10.3205/zma001680
Improv practices in Mathematics Active Teaching	Morales	2022	USA	Mathematics	Teachers	No	Programme /8 weeks	Communication, collaboration, error management, active listening	/		Yes	Not mentionned	Morales-Almazan, P. (2022). Improv Practices in Mathematics Active Teaching. Primus: Problems, Resources & Issues in Mathematics Undergraduate Studies, 32(9), 1013-1028. afh.
Improv comedy and modern marketing education : Exploring consequences for divergent thinking, self-efficacy, and collaboration.	Mourey	2019	USA	business, medicine, nursing, education	Students	Yes	Programme/10 weeks for study 2	interprofessional collaboration, divergent thinking	Divergent thinking test<br>Creative marketing task (Oreo)<br>Self-evaluation scales	At 4 months	Yes (in the annex)	University teacher	Mourey, J. A. (2020). Improv comedy and modern marketing education : Exploring consequences for divergent thinking, self-efficacy, and collaboration. Journal of Marketing Education, 42(2), 134-148. psyh. https://doi.org/10.1177/0273475318822087
The impact of a medical improv curriculum on wellbeing and professional development among pre-clinical medical students	Neel	2021	USA	medicine	Students	No	Programme/9 weeks (weekly sessions of 2 hours)	Communication, Self-confidence, Interpersonal relations, Well-being	Questionnaires with Likert scale (1-5) and qualitative opens question	Pre/post	Not completely	Two instructors trained in improvisation	Neel, N., Maury, J.-M., Heskett, K. M., Iglewicz, A., & Lander, L. (2021). The impact of a medical improv curriculum on wellbeing and professional development among pre-clinical medical students. Medical Education Online, 26(1), 1961565. https://doi.org/10.1080/10872981.2021.1961565
Embodying improvisational education for managers: learning from theater	Paquelet	2022	Bresil	management	Students	No	Programme/6 academic sessions	Adaptability, Interpersonal relations	Direct observation<br>Group semi-structured interviews	During/post	Not completely	A teacher with theater training, and a management teacher	Paquelet Moreira, B. F., Davel, E., & Cunha, M. P. e. (2022). Embodying improvisational education for managers : Learning from theater. Culture & Organization, 28(3/4), 296-312. afh.
Fostering psychological safety: Using improvisation as a team building tool in management education	Perrmann	2022	USA	management	Students	No	One-off workshop/3 hours	Self-confidence, collaboration, creativity, risk-taking	Individual written reflections<br>Analysis of Q&A discussions	During/post	Yes	University improv troupe	Perrmann-Graham, J., Liu, J., Cangioni, C., & Spataro, S. E. (2022). Fostering psychological safety : Using improvisation as a team building tool in management education. International Journal of Management Education (Elsevier Science), 20(2), N.PAG-N.PAG. bth. https://doi.org/10.1016/j.ijme.2022.100617
Improvisation as a Teaching Tool for Improving Oral Communication Skills in Premedical and Pre-Biomedical Graduate Students	Phelps	2021	USA	medicine, biomedical sciences	Students, New graduates	No	One-off workshop/3 hours	Communication, Self-confidence, Empathy, Creativity	Empathy Quotient (self-report questionnaire)<br>Online questionnaire (33 items)	During/post	Yes<br>"Yes, And"<br>Passage of imaginary objects<br>Group machine<br>Mirror<br>Questions and answers	Instructor with advanced training in theater	Phelps, M., White, C., Xiang, L., & Swanson, H. I. (2021). Improvisation as a Teaching Tool for Improving Oral Communication Skills in Premedical and Pre-Biomedical Graduate Students. Journal of Medical Education and Curricular Development, 8, 23821205211006411. https://doi.org/10.1177/23821205211006411
Learning Science Communication Skills Using Improvisation, Video Recordings, and Practice, Practice, Practice	Ponzio	2018	USA	multidisciplinary	Doctoral students	No	Programme/ 15 weeks, weekly sessions of 3 hours	Communication, Speaking	Evaluation grid for video<br>Satisfaction questionnaire	Pre/post	Not completely	Multidisciplinary team (sciences, communication, theater)	Ponzio, N. M., Alder, J., Nucci, M., Dannenfelser, D., Hilton, H., Linardopoulos, N., & Lutz, C. (2018). Learning Science Communication Skills Using Improvisation, Video Recordings, and Practice, Practice, Practice. Journal of Microbiology & Biology Education, 19(1), 19.1.15. https://doi.org/10.1128/jmbe.v19i1.1433
The Benefits of Improvisational Games in the TC Classroom	Rice-Bailey	2020	USA	multidisciplinary	Students	No	One-off workshop/40 minutes	Adaptability, Self-confidence, Collaboration, Creativity	Satisfaction questionnaire (Likert and open questions)	Post (1 week after)	Yes (in the annex)	One instructor trained in improvisation	Rice-Bailey, T. (2021). The Benefits of Improvisational Games in the TC Classroom. Technical Communication Quarterly, 30(1), 63-76. afh.
Teaching Yes, And... Improv in Sales Classes: Enhancing Student Adaptive Selling Skills, Sales Performance, and Teaching Evaluations	Rocco	2014	USA	business, marketing	Students	Yes	Programme/4 weeks, 15 hours total	Adaptability	Satisfaction questionnaire (Likert scale 1-5 points)	Post	Yes<br>"Yes and"	Professor and sales executives	Rocco, R. A., & Whalen, D. J. (2014). Teaching Yes, And...Improv in sales classes : Enhancing student adaptive selling skills, sales performance, and teaching evaluations. Journal of Marketing Education, 36(2), 197-208. psyh. https://doi.org/10.1177/0273475314537278
"Just what is there now, that is what there is"—the effects of theater improvisation training on clinical social workers' perceptions and interventions	Romanelli	2019	Israel	clinical social work	Students	Yes	Programme/1 semester	Adaptability, Interpersonal relations, Collaboration	DPCCQ (Development of Psychotherapist Common Core Questionnaire)<br>WAI-SR (Working Alliance Inventory-Short Form Revised) TPI-T (Therapist Presence Inventory)<br>Semi-structured interviews	Pre/Post/ at 3 months<br>Interviews at 3 months	Not completely	1rst author (social worker, family therapist and improvisation trainer)	Romanelli, A., & Tishby, O. (2019). « Just what is there now, that is what there is'—The effects of theater improvisation training on clinical social workers » perceptions and interventions. Social Work Education, 38(6), 797-814. afh.
"Coming home to myself": A qualitative analysis of therapists' experience and interventions following training in theater improvisation skills	Romanelli	2017	Israel	clinical social work	Students, New graduates	No	Programme/1 semester	Adaptability, Self-confidence, Emotions, Interpersonal relations	Semi-structured interviews	Post at 3-4 months	No	Not mentionned	Romanelli, A., Tishby, O., & Moran, G. S. (2017). "Coming home to myself" : A qualitative analysis of therapists' experience and interventions following training in theater improvisation skills. Arts in Psychotherapy, 53, 12-22. afh.
Exploring the Value of Improvisational Theater in Medical Education for Advancing the Doctor–Patient Relationship and Health Equity	Rusiecki	2023	USA	medicine	Students	No	One-off virtual workshop/90 minutes	Adaptability, Communication, Empathy, Active listening	Satisfaction questionnaire (Likert and open questions)<br>Structured interviews	Post	Yes<br>Group Mirroring<br>Yes Circle	Doctor, scientist, professional improviser	Rusiecki, J. M., Orlov, N. M., Dolan, J. A., Smith, M. P., Zhu, M., & Chin, M. H. (2023). Exploring the Value of Improvisational Theater in Medical Education for Advancing the Doctor-Patient Relationship and Health Equity. Academic Medicine: Journal of the Association of American Medical Colleges, 98(6S), S46-S53. https://doi.org/10.1097/ACM.0000000000005183
Safe and Sound: An Improvisational Theater-Based Curriculum and Behavioral Intervention to Address Violence in the Emergency Department	Sanky	2023	USA	medicine	Students	No	Programme/several months (June-December)	Conflict management, Stress management, Well-being	Connor-Davidson Resilience Scale (adapted)<br>Satisfaction questionnaire (Likert and open questions)	Pre/Post/ at 3 months	Yes (in the annex)	Not mentionned	Sanky, C. (2023). Safe and Sound : An Improvisational Theater-Based Curriculum and Behavioral Intervention to Address Violence in the Emergency Department. The Journal of Emergency Medicine, 64(2), 236-245. https://doi.org/10.1016/j.jemermed.2022.11.002
Substantial Increases in Healthcare Students' State Empathy Scores Owing to Participation in a Single Improvisation Session	Schwartz	2024	USA	medicine, biomedical sciences, health sciences	Students	No	One-off workshop/one to two hours	Empathy	State Empathy Scale adapted (12 items)	Pre/Post	Yes<br>Mirror games<br>Storytelling in pairs<br>"Yes, let's!" exercise<br>Game "Dr. Know-it-all"	Faculty member with experience in improvisation	Schwartz, B. D., Rogers, S. L., Michels, N., & Van Winkle, L. J. (2024). Substantial Increases in Healthcare Students' State Empathy Scores Owing to Participation in a Single Improvisation Session. International Journal of Environmental Research and Public Health, 21(5), 531. https://doi.org/10.3390/ijerph21050531
The Paradox of Fiction Revisited—Improvised Fictional and Real-Life Social Rejections Evoke Associated and Relatively Similar Psychophysiological Responses	Seppänen	2021	Finland	education	Students	Yes	One-off workshop/ 2h30	Emotions, Stress management, Interaction with others	Psychophysiological measures:<br>ECG (heart rate)<br>EDA (electrodermal activity)<br>Facial EMG (muscle activity)<br>EEG (brain activity)<br><br>Self-assessment of stress (Likert scale 0-5)	During (psychophysiological measures)<br><br>Post (self-assessment)	No	Researcher specializing in theatrical improvisation	Seppänen, S., Toivanen, T., Makkonen, T., Jääskeläinen, I. P., & Tiippana, K. (2021). The Paradox of Fiction Revisited-Improvised Fictional and Real-Life Social Rejections Evoke Associated and Relatively Similar Psychophysiological Responses. Brain Sciences, 11(11), 1463. https://doi.org/10.3390/brainsci11111463
Thinking on my feet': an improvisation course to enhance students' confidence and responsiveness in the medical interview	Shochet	2013	USA	medicine	Students	No	Programme/4 weeks, weekly sessions of 2 hours	Active listening, Self-confidence, Collaboration, Communication, Flexibility	Satisfaction questionnaire (Likert and open questions)<br>Group discussion	Post	Yes<br>"So what you are saying"<br>"Yes, and..."	Clinical teachers and trainers in theatrical improvisation	Shochet, R., King, J., Levine, R., Clever, S., & Wright, S. (2013). 'Thinking on my feet': An improvisation course to enhance students' confidence and responsiveness in the medical interview. Education for Primary Care, 24(2), 119-124. https://doi.org/10.1080/14739879.2013.11493466
Ethical Challenges in the Teaching of Improvisation for Psychologists' Communication	Temezhnikova	2020	Russia	psychology	Students	No	Programme/2 sessions with a week's preparation in between	Stress management, communication, spontaneity	In house evaluation cotation	During / Post	No	Teacher-researchers in psychology	Temezhnikova, O. B., & Bazarov, T. Yu. (2020). Ethical challenges in the teaching of improvisation for psychologists' communication. Psychology in Russia: State of the Art, 13(2), 154-164. psyh. https://doi.org/10.11621/pir.2020.0211
Development of an empathy and clarity rating scale to measure the effect of medical improv on end-of-first-year OCSE performance: a pilot study	Terregino	2019	USA	Medicine	Students	Yes	Standard training: approximately 3 hours + Enhanced training: an additional 6 hours	Communication, Empathy, Interpersonal relations	ECRS (Empathy and Clarity Rating Scale)	During OCSE	No	Not mentionned	Terregino, C. A., Copeland, H. L., Sarfaty, S. C., Lantz-Gefroh, V., & Hoffmann-Longtin, K. (2019). Development of an empathy and clarity rating scale to measure the effect of medical improv on end-of-first-year OSCE performance: A pilot study. Medical Education Online, 24(1), 1666537. https://doi.org/10.1080/10872981.2019.1666537
Connecting improvisational exercises and pharmacy communication skills: A how-to guide	Tetenbaum-Novatt	2023	USA	Pharmacy	Students	No	Programme/3 one-hour sessions over a six-month period	Communication, interaction with others	Satisfaction questionnaire<br>Observation during OCSE	During OCSE	Yes	Teacher with theatrical experience	Tetenbaum-Novatt, J. E., & Alexander, A. J. (2023). Connecting improvisational exercises and pharmacy communication skills : A how-to guide. Currents in Pharmacy Teaching & Learning, 15(2), 201-210. https://doi.org/10.1016/j.cptl.2023.02.027
Teaching Reference Interview Skills with Improv	Vardell	2022	USA	Library science	Students	No	Programme/1h30 per session (number of sessions not specified)	Communication, flexibility, active listening	Satisfaction questionnaire	Post	Yes<br>One Word Story<br>Alphabet Game	Teachers	Vardell, E., & Nelson, S. B. (2022). Teaching Reference Interview Skills with Improv. Journal of Education for Library & Information Science, 63(1), 38-56. afh.
"Experiencing complex stakeholder dynamics around emerging technologies: a role-play simulation"	Visscher	2023	Netherland	engineer	Students	No	One-off workshop/2h	uncertainty management	Satisfaction questionnaire	Post	Not completely	Not mentionned	Visscher, K. (2024). Experiencing complex stakeholder dynamics around emerging technologies : A role-play simulation. European Journal of Engineering Education, 49(2), 370-388. afh.
How to catch a flying pig: facilitating embodiment work in online rooms	Vuuren	2020	South-Africa and Autrich	multidisciplinary	Doctoral students,Teachers	No	Programme/Multiple sessions over an unspecified period	learning	Feedback	During	Yes<br>Sound Ball<br>Mirror Game<br>Walking Exercise<br>Pig Catching Signature Move	Experienced improvisation facilitators	Vuuren, P. J. van, & Freisleben, C. F. (2020). How to catch a flying pig : Facilitating embodiment work in online rooms. Research in Drama Education, 25(2), 268-285. afh.
Serious Play: Teaching Medical Skills With Improvisational Theater Techniques	Watson	2011	USA	medicine	Students	No	Programme/5 weekly sessions of 2 hours, total of 10 hours	Adaptability,Communication,Self-confidence,Stress management,Collaboration,Active listening	Satisfaction questionnaire (Likert and open questions)	Post	Not completely	Teacher trained in improvisation and professor of medical ethics	Watson, K. (2011). Serious play : Teaching medical skills with improvisational theater techniques. Academic Medicine, 86(10), 1260-1265. psyh. https://doi.org/10.1097/ACM.0b013e31822cf858
"Off-Script, Online: Virtual Medical Improv Pilot Program for Enhancing Well-being and Clinical Skills among Psychiatry Residents"	Westcott	2023	Canada	psychiatry	Students	No	Programme/5 weekly sessions of 90 minutes (March-April 2021) + 1 'teaser' session in February 2021	Communication,Conflict management,well-being,collaboration	Recorded debriefings<br>Focus group	Post / At 1 month (focus group)	Yes	Medical improvisation facilitators	Westcott, S., Simms, K., van Kampen, K., Jafine, H., & Chan, T. M. (2023). Off-Script, Online : Virtual Medical Improv Pilot Program for Enhancing Well-being and Clinical Skills among Psychiatry Residents. Academic Psychiatry: The Journal of the American Association of Directors of Psychiatric Residency Training and the Association for Academic Psychiatry, 47(4), 374-379. https://doi.org/10.1007/s40596-023-01778-6
Interprofessional Improv: Using Theater Techniques to Teach Health Professions Students Empathy in Teams	Zelenski	2020	USA	multidisciplinary	Students	No	Programme/6 sessions of 2h30 over 6-8 weeks, total of 15 hours	Communication, Emotions, Empathy, Interaction with others	Interpersonal Reactivity Index (IRI)<br>Consultative and Relational Empathy (CARE) measure Facial expression recognition test<br>Semi-structured interviews	Pre/post	Not completely	Experienced in applied improvisation facilitators	Zelenski, A. B., Saldivar, N., Park, L. S., Schoenleber, V., Osman, F., & Kraemer, S. (2020). Interprofessional Improv : Using Theater Techniques to Teach Health Professions Students Empathy in Teams. Academic Medicine: Journal of the Association of American Medical Colleges, 95(8), 1210-1214. https://doi.org/10.1097/ACM.0000000000003420
Student teachers' experience with improvisation activities for spontaneous speech practice in English	Zondag	2021	Norway (Nord University)	English as a Foreign Language (TEFL)	Students, teachers	No	Programme/3 sessions of 1 hour	Communication, Self-confidence, Stress management, Interaction with others, Creativity	Semi-structured interviews<br>Feedback	During/post	Yes	Trainer with dual skills (improvisation and teaching)	Zondag, A. (2021). Student teachers' experience with improvisation activities for spontaneous speech practice in English. Language Teaching Research, 1. afh.
