# Supplementary material for: The use of applied improvisation at university: a mini-review
Source: Front Psychol. 2026 Jan 7;16:1661912. doi: 10.3389/fpsyg.2025.1661912 (PMC12819688; doi:10.3389/fpsyg.2025.1661912)
Supplement: Supplementary file 2 [file Data_Sheet_2.PDF]

Supplementary Table 2

Distribution of target populations and intervention formats across disciplines in applied improvisation programs (N=54 studies)

Disciplinary domain	Number of studies (%)	Target population	Format	References
**Health - Medicine**	27 (50%)	Medical students (all levels)	Long intervention (n=14)<br>Single workshop (n=12)<br>Not mentioned (n=1)	(Amjadi et al., 2024; Ayub et al., 2024; Ballon et al., 2007; Bender et al., 2022; Bing-You et al., 2018; Cai et al., 2019; De Carvalho Filho et al., 2020; De Wever, et al., 2023; Erdman & Dellasega, 2024; Fessell et al., 2020; Grossman et al., 2021; Hammer et al., 2011; Hobson et al., 2019; Hoffman et al., 2008; Kaplan-Liss et al., 2018; Kukora et al., 2020; Li et al., 2022; Minow et al., 2024; Mourey, 2020; Neel et al., 2021; Phelps et al., 2021; Rusiecki et al., 2023; Sanky, 2023; Schwartz et al., 2024; Shochet et al., 2013; Terregino et al., 2019; Watson, 2011)
**Health - Pharmacy**	3 (6%)	Pharmacy students	Long intervention (n=2)<br>Single workshop (n=1)	(Boesen et al. 2009; Donovan et al. 2020; Tetenbaum-Novatt & Alexander 2023)
**Health - Nursing**	5 (9%)	Nursing students	Long intervention (n=4)<br>Single workshop (n=1)	(Bender et al., 2022; De Wever, Hainselin, et al., 2023; Del Vecchio et al., 2022; Kaplan-Liss et al., 2018; Mourey, 2020)
**Health - Other disciplines**	8 (15%)	Speech therapy (n=2)<br>Genetic counseling (n=1)<br>Biomedical sciences (n=2)<br>Psychiatry (n=1)<br>Paediatrics (n=1)<br>Health sciences (n=1)	Long intervention (n=2)<br>Single workshop (n=5)<br>Not mentioned (n=1)	(Bender et al., 2022; De Wever, et al., 2023; De Wever, et al., 2023; Hoffmann-Longtin et al., 2018; Li et al., 2022; Phelps et al., 2021; Schwartz et al., 2024; Westcott et al., 2023)
**Management/Business**	5 (9%)	Business (n=2)<br>Management students (n=3)	Long intervention (n=4)<br>Single workshop (n=1)	(Huffaker & West, 2005; Mourey, 2020; Paquelet Moreira et al., 2022; Perrmann-Graham et al., 2022; Rocco & Whalen, 2014)
**Education**	4 (7%)	Mathematics (n=1)<br>Education (n=2)<br>TEFL (English class) (n=1)	Long intervention (n=3)<br>Single workshop (n=1)	(Morales-Almazan, 2022; Mourey, 2020; Seppänen et al., 2021; Zondag, 2021)
**Clinical social work**	2 (4%)	Social work students	Long intervention	(Romanelli & Tishby 2019; Romanelli et al. 2017)

**Various disciplines**	5 (9%)	Engineering (n=1)<br>Library science (n=1)<br>Psychology (n=1)<br>Sports (STAPS) (n=1)<br>Consulting (n=1)	Long intervention (n=3)<br>Single workshop (n=2)	(Archieri, 2022; Lawrence & Coaston, 2017; Temezhnikova & Bazarov, 2020; Vardell & Nelson, 2022; Visscher, 2023)
**Mixed disciplinary**	12 (22%)	Health disciplines (n=6)<br>Business & marketing (n=1)<br>Multidisciplinary (n=5)	Long intervention (n=6)<br>Single workshop (n=5)	(Bender et al., 2022; De Wever, et al., 2023; Kaplan-Liss et al., 2018; Li et al., 2022; Mourey, 2020; Phelps et al., 2021; Ponzio et al., 2018; Rice-Bailey, 2021; Rocco & Whalen, 2014; Schwartz et al., 2024; Vuuren & Freisleben, 2020; Zelenski et al., 2020)
**Mixed populations**	7 (13%)	Students & medical staff (n=2)<br>Students & new graduates (n=3)<br>Students & teachers (n=2)	Long intervention (n=5)<br>Single workshop (n=2)	(Ayub et al., 2024; Ballon et al., 2007; Li et al., 2022; Phelps et al., 2021; Romanelli et al., 2017; Vuuren & Freisleben, 2020; Zondag, 2021)

Note: A long intervention is more than one session, while a single workshop is just one session. We were unable to calculate an average intervention duration due to the wide variation in interventions lengths (either in hours or weeks).
